# Compressive stenosis of the left hepatic vein as a pathogenesis of postresectional liver failure: a case report

**DOI:** 10.1186/1752-1947-4-163

**Published:** 2010-05-28

**Authors:** Mizuki Ninomiya, Tetsuo Ikeda

**Affiliations:** 1 Department of Surgery, Oita Prefectural Hospital, Bunyo, Oita, 870-8511, Japan; 2 Department of Surgery, National Hospital Organization Fukuoka Higashi Medical Center, Koga, 811-3195, Japan

## Abstract

**Introduction:**

Postresectional liver failure (PLF) is a devastating and fatal complication of major hepatic resection, and we do not have a full understanding of the pathogenic mechanisms involved. No reliable treatment other than liver transplantation currently exists for PLF.

**Case presentation:**

A 46-year-old Japanese man experienced PLF after an extended right hepatectomy for liver malignancy. Seven months after surgery, the patient's Model for End-Stage Liver Disease (MELD) score had reached 23. Doppler ultrasound study and three-dimensional computed tomography images showed a stenosed left hepatic vein compressed by surrounding hypertrophied hepatic parenchyma. Transluminal balloon angioplasty and stent placement therapy were conducted eight months after surgery. The pressure gradient between the hepatic vein and right atrium decreased from 13 to 3 mmHg after stent placement. Thereafter, the patient recovered.

**Conclusion:**

Hepatic venous compression by surrounding hypertrophied hepatic parenchyma might, at least in part, be associated with the occurrence of PLF. Surgeons should bear this possibility in mind when confronted with cases of PLF, as early diagnosis and stent placement improves patients' chances of recovery.

## Introduction

Hepatic resection is the preferred treatment for hepatic malignancies like hepatocellular carcinoma or colorectal liver metastases. Although major hepatic resection is now accomplished with mortality rates of less than 5% in high volume centers, post-resectional liver failure (PLF) remains a potentially devastating complication and often proves fatal [1]. The risk of PLF increases as the amount of resected liver parenchyma increases. In general, the relative remnant liver volume (RLV) ratio, defined as the percentage remaining liver volume compared with standard liver volume, is regarded as a reliable parameter in the prediction of PLF. In normal livers, an RLV ratio below 25% has been reported to be a strong predictor of serious hepatic dysfunction following liver resection [2,3]. Unfortunately, no reliable disease-specific therapy exists for PLF, with the exception of liver transplantation in some limited cases, and PLF mortality rates are between 60 and 80% [4,5]. Understanding the exact pathophysiology of PLF would enable us to establish effective treatments other than liver transplantation. Here we present a case of PLF that was successfully treated by hepatic venous stent placement therapy. We also discuss the possible pathogenesis of PLF.

## Case presentation

A 46-year-old Japanese man was referred to our hospital with intrahepatic cholangiocellular carcinoma. The tumor was 3 cm in diameter and located at segment 8, between the root of the middle and right hepatic veins. Preoperative liver function tests were normal, and preoperative blood work was negative for hepatitis virus markers. The patient underwent an extended right hepatectomy without resection of the extrahepatic bile duct. The patient's middle hepatic vein was divided approximately 2 cm upstream from the root of the middle and left hepatic veins.

The patient's postoperative course was uneventful until day 10, when a gradual increase in serum bilirubin levels and ascites formation were noted. Although obstructive jaundice and portal thrombosis were excluded by ultrasound and computed tomography (CT) scans, the patients continued to deteriorate, displaying hyperbilirubinemia, coagulopathy and refractory ascites (Table 1). Neither circulatory disturbance nor hepatic encephalopathy were seen during this period. The patient's Model for End-Stage Liver Disease (MELD) score also increased gradually, and reached 23 seven months after the initial hepatic surgery. MELD scores between 20 and 29 are associated with 3-month mortality rates of over 75%. Repeated abdominal paracentesis were necessary to treat his refractory ascites, which amounted to more than 10,000 ml per week.

**Table 1:** Parameters of liver function before and after operation

	Preoperative	Postoperative		
		
		1 month	4 months	7 months
Total bilirubin (mg/dl)	1.2	9.8	16.5	30.1

Albumin (g/dl)	4.4	3.2	2.8	2.3

Prothorombin time (%)	112	89	81	68

AST (U/dl)	30	48	46	51

MELD score	-	13	14	23

AST, aspartate aminotransferase; MELD, model for end-stage liver disease

**Figure 1:**
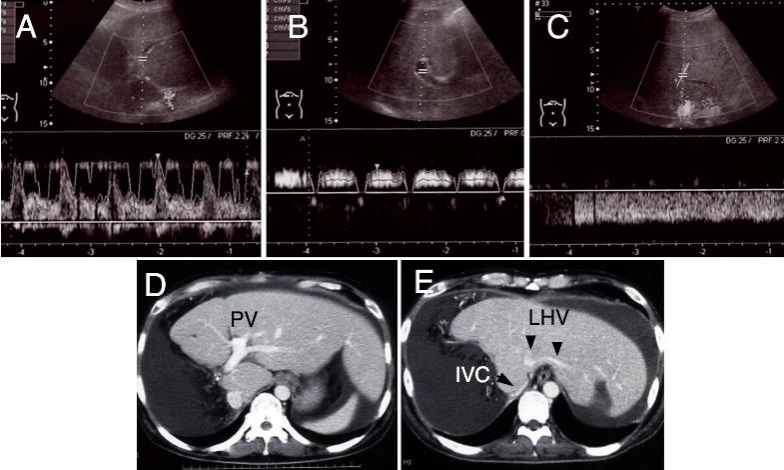
**Spectral Doppler ultrasound study and contrast-enhanced computed tomography (CT) images at 7 months after surgery**.

**Figure 2:**
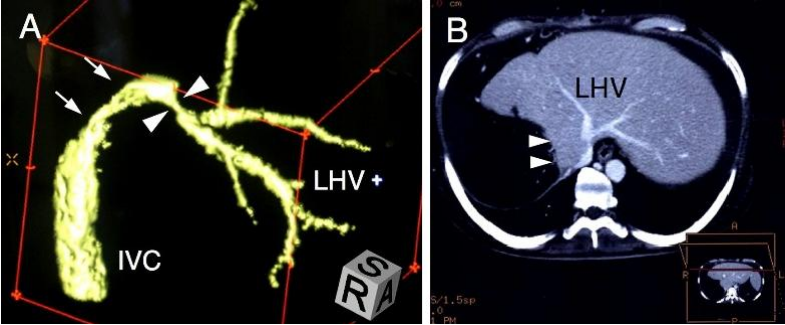
**Images from computed tomography (CT) data delineating the stenosis of hepatic vein**.

In order to rule out an insufficient hepatic regeneration, the patient's liver volume was assessed by CT-volumetry using previously described methods [6]. Seven months after surger, his liver volume was 1290 ml, almost identical to the standard liver volume (1222 ml) which was calculated using Urata's formula [7]. Nevertheless, serum levels of hepatocyte growth factor were elevated to 1.61 ng/ml, signifying that the liver was still being stimulated to regenerate. Serum hyaluronic acid levels were also elevated to 3030 ng/ml, suggesting that sinusoidal endothelial cell function was severely disturbed [8]. An assessment of the patient's hepatic circulation by spectral Doppler ultrasound revealed a good hepatic arterial flow, but a relatively weak intrahepatic portal signal with no diastolic flow. The Doppler waveform of hepatic venous flow was monophasic, suggesting the presence of hepatic venous stenosis (Figure 1). Because axial CT images only were not conclusive regarding the presence of hepatic vein stenosis, multiplanar reconstruction (MPR) images and three-dimensional CT images were also assessed, and the stenosis at the root of the left hepatic vein was clearly visualized (Figure 2).

To further confirm the presence of hepatic venous stenosis, the pressure gradient between the left hepatic vein and right atrium was measured. Hepatic venous catheterization was performed through the jugular vein. The mean hepatic venous pressure was 15.6 mmHg, and the pressure abruptly decreased when the catheter was pulled through the assumed stenotic point into the right atrium. The pressure gradient between the left hepatic vein right atrium was 13.2 mmHg. The normal hepatic venous pressure gradient lies between 1 and 2 mmHg [9]. Thus, we confirmed the presence of hepatic venous stenosis morphologically and functionally.

Written informed consent was obtained from the patient for endovascular treatment. As an initial treatment option, hepatic venous balloon dilation was performed eight months after surgery. A transluminal angioplasty catheter (Synergy Balloon Catheter, Boston Scientific, Tokyo) with a balloon diameter of 10 mm and a length of 40 mm was used for venous dilatation. Although the pressure gradient across the left hepatic vein and right atrium decreased from 13 to 4 mmHg immediately after intervention, the effect was temporary. Ten minutes after balloon dilatation, the pressure gradient had increased to 10 mmHg. Although serum bilirubin levels decreased unexpectedly to near the normal range, refractory ascites remained. Therefore, the patient underwent endovascular stent placement therapy seven weeks later.

**Figure 3:**
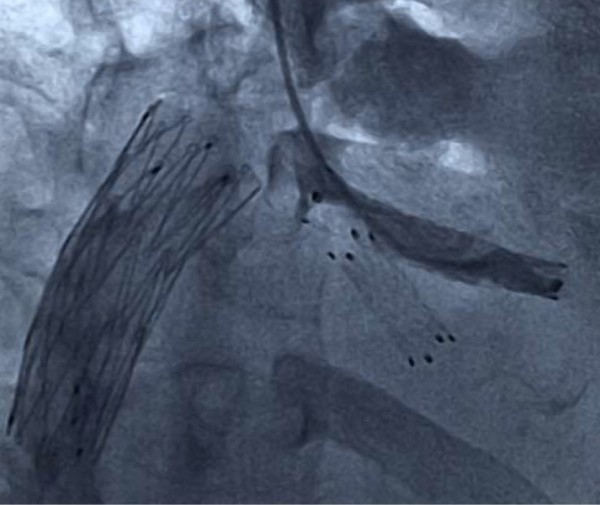
**Hepatic venography after stent placement**.

**Figure 4:**
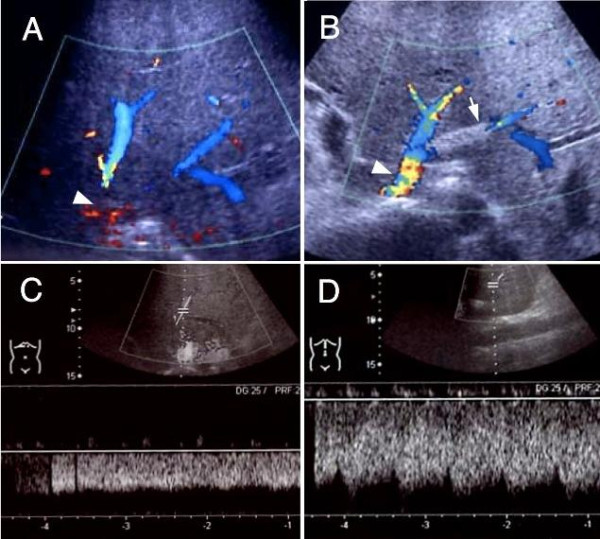
**The Doppler ultrasound images before (A, C) and after (B, D) the stent placement therapy**.

A self-expandable metallic stent (Luminexx, BARD, Covington, GA) with a diameter of 8 mm and a length of 40 mm was placed into the left hepatic vein via the internal jugular vein. Because the large umbilical fissure vein was bifurcated just behind the stenosis, another metallic stent with a diameter of 8 mm and a length of 2 mm was inserted into it using a stent-in-stent technique (Figure 3). At the same time, in order to relieve the symptom of lower leg edema, the retrohepatic inferior vena cava was also dilated with a metallic stent (Spiral Z stent, Wilson-Cook Inc., Winston-Salem, MA) with a diameter of 20 mm and a length of 60 mm. The pressure gradient across the left hepatic vein and right atrium decreased to 3 mmHg just after stent placement, which was maintained even after 10 minutes. A Doppler ultrasound study three weeks after stent placement showed an increased hepatic venous flow velocity and a pulsatile venous waveform (Figure 4). Along with the normalized bilirubin level, the patient's ascitic fluid volume also decreased favorably after the stent placement. Thus, the patient had recovered from his PLF.

## Discussion

Although many factors have been proposed to be associated with increased risk of PLF, including inadequate hepatic regeneration, pre-existing cirrhosis and prolonged liver ischemia during resection, the critical factor is believed to be an insufficient remnant liver mass [3]. Despite this, the mechanisms of PLF in the majority of clinical cases are thought to be multifactorial, not due purely to small remnant liver volume.

An adequate hepatic venous drainage is a prerequisite in the recovery process of a damaged liver and its significance is magnified in the case of extended right hepatectomy or trisegmentectomy, whereby the left hepatic vein is the only conduit to drain the entire remaining liver. It seemed that in our case, the root of the left hepatic vein was compressed by hypertrophied liver parenchyma subsequent to a vigorous regenerative response. Although reports of postoperative hepatic venous stenosis are common, most of these are stenosis after liver transplantation, whereby hepatic veins are more prone to mechanical stenosis than in the case of hepatectomy. This is due to suture anastomosis and to the possibility of venous kinking associated with graft dislocation [10]. It could be inferred that hepatic venous compression by surrounding hypertrophied liver parenchyma might have been overlooked as the pathogenesis of PLF in previous cases. The diagnosis of hepatic venous stenosis by means of ordinary CT images seemed to be much more difficult than expected, because a single axial CT image could not clearly depict the outline of the hepatic vein with caudal inclination. Meanwhile, with the spread of liver transplantation, the usefulness of Doppler ultrasound for the diagnosis of hepatic venous stenosis had been calrified. Ko *et al. *reported that a persistent monophasic wave pattern on Doppler ultrasound images suggested, but did not conclusively indicate, hepatic venous stenosis after liver transplantation [11]. Therefore, when hepatic venous stenosis is suspected as a cause of PLF, a screening Doppler ultrasound study should be used to assist in making a definitive diagnosis, taking into consideration other studies such as three-dimensional CT imaging, hepatic venography and measurement of the pressure gradient across the stenosis.

**Figure 5:**
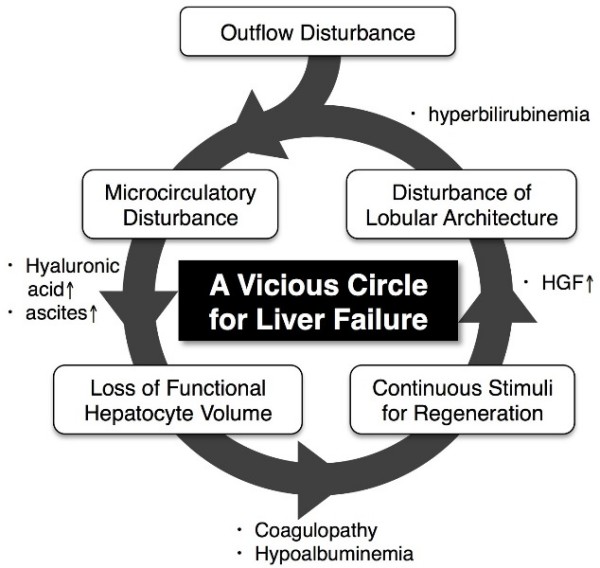
**The possible mechanism of postresectional liver failure in the present case**.

Figure 5 diagrams the probable mechanism of PLF in our case. Outflow disturbance of the liver had led to a microcirculatory disturbance, and subsequently to a decrease in the functional hepatocyte volume. In order to meet the metabolic demand under these circumstances, the liver might have been stimulated chronically to regenerate, as represented by elevated hepatocyte growth factor levels in the serum. Hepatic regeneration is accompanied by a complex remodeling of the hepatic tissue with a transient breakdown of the lobular architecture. As we reported previously, the more the liver is stimulated to regenerate, the greater the derangement of lobular architecture and consequently hyperbilirubinemia and microcirculatory disturbances [12]. Such a vicious circle for PLF might have been disconnected by hepatic venous stent placement, ameliorating microcirculatory disturbances.

Transluminal balloon angioplasty and stent placement therapy are currently the preferred mode of treatment for hepatic venous stenosis. According to the treatment results reported by Ko *et al.*, hepatic venous stenoses after liver transplantation were treated favorably by balloon angioplasty, although repeat angioplasty was necessary against restenosis [13]. The main cause of anastomotic stenosis after liver transplantation is thought to be a fibrosis or intimal hyperplasia around the anastomotic site. However, stenosis after partial hepatectomy seems to be attributable, at least in part, to compression by surrounding hypertrophied parenchyma. Therefore, as in the present case, the treatment effects of balloon angioplasty for compressed stenosis are temporary and limited. From our experience of the present case, we thought that stent placement would be a preferable treatment measure for stenosis caused by extrinsic compression.

## Conclusions

Hepatic venous compression by surrounding hypertrophied hepatic parenchyma might, at least in part, be associated with the occurrence of PLF. Recognition of hepatic venous compression as one of the pathogenic mechanisms of PLF may help to establish an adequate mode of treatment, such as stent placement therapy, and improve the prognosis of patients without requiring liver transplantation.

## Abbreviations

CT: computed tomography; MELD: Model for End-Stage Liver Disease; MPR: multiplanar reconstruction; PLF: postresectional liver failure; RLV: relative remnant liver volume.

## Consent

Written informed consent was obtained from the patient's family for the publication of this case report and any accompanying images. A copy of the written consent is available for review by the Editor-in-Chief of this journal.

## Competing interests

The authors declare that they have no competing interests.

## Authors' contributions

MN interpreted the patient's data, devised the therapeutic plan, and wrote the manuscript. TI helped in planning the therapeutic plan and drafting the manuscript. All authors read and approved the final manuscript.
